# Supplementary material for: Generation of an Adequate Perfusion Network within Dense Collagen Hydrogels Using Thermoplastic Polymers as Sacrificial Matrix to Promote Cell Viability
Source: Bioengineering (Basel). 2022 Jul 14;9(7):313. doi: 10.3390/bioengineering9070313 (PMC9311520; doi:10.3390/bioengineering9070313)
Supplement: Supplementary file 1 [file bioengineering-09-00313-s001.zip › bioengineering-1805090-supplementary.pdf]

Supplementary information

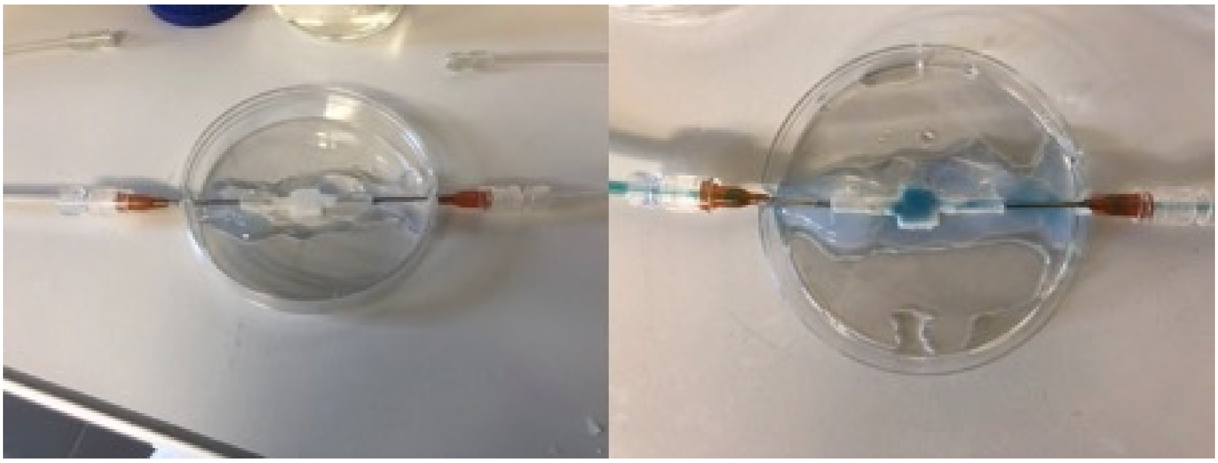

Figure S1. *Perfusion setup. Gels were glued inside a petri dish using Agarose 5% wt. Flat bottom needles (15G) were inserted in the gel extremities and were connected to the syringe controller. Gels were perfused at 0.05 mL·min*$^{-1}$.

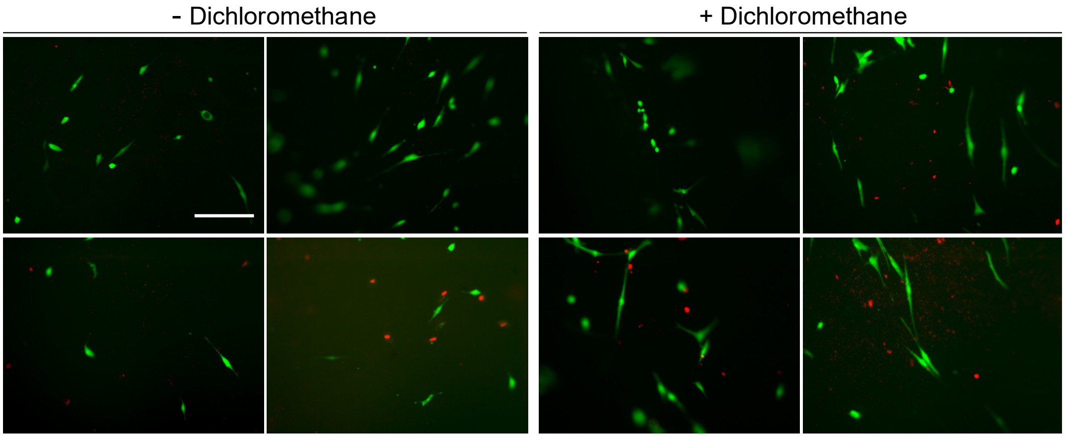

Figure S2. *NHDFs were cultivated on top of collagen gels for 24h and colored with a Live (green)/Dead (red) staining. (Scale bar 250 µm).*

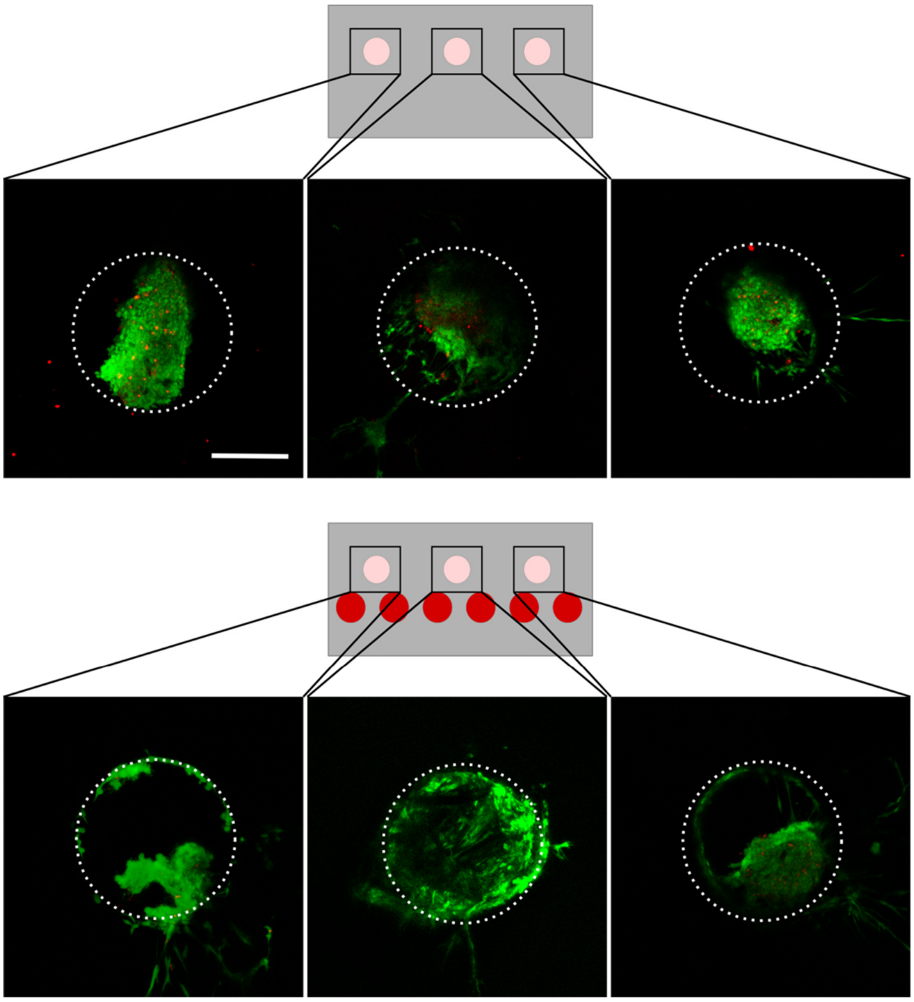

Figure S3. *Cell survival in the middle of the gel for the three channels. NHDFs were cultivated for one month before being analyzed with a Live/Dead staining. Scale bar 250 µm*
